# Bibliometric and visual analysis in the field of ketogenic diet on cancer from 2012 to 2021

**DOI:** 10.3389/fnut.2022.1060436

**Published:** 2022-11-10

**Authors:** Rongrong Li, Qingcheng Huang, Chenxiao Ye, Changhong Wu, Ning Luo, Yi Lu, Jianqiao Fang, Yun Wang

**Affiliations:** ^1^The Third Affiliated Hospital of Zhejiang Chinese Medical University, Hangzhou, China; ^2^First Clinical College of Zhejiang Chinese Medical University, Hangzhou, China; ^3^Second Clinical College of Zhejiang Chinese Medical University, Hangzhou, China; ^4^Third Clinical College of Zhejiang Chinese Medical University, Hangzhou, China; ^5^Department of Clinical Nutrition, The Cancer Hospital of the University of Chinese Academy of Sciences (Zhejiang Cancer Hospital), Institute of Basic Medicine and Cancer, Chinese Academy of Sciences, Hangzhou, China; ^6^Department of Oncology, The First Affiliated Hospital of Zhejiang Chinese Medical University, Hangzhou, China

**Keywords:** bibliometric analysis, ketogenic diet, cancer, mechanism, immunotherapy, clinical trials

## Abstract

Increasing evidence demonstrated that the ketogenic diet (KD) played a positive effect on cancer treatment. However, no systematic review and bibliometric analysis were conducted in this field. This study aimed to explore the current status, and reveal the potential trends and hotspots to provide a reference for future research. Publications were extracted from the Web of Science Core Collection. CiteSpace (5.6.R3) software and the website of bibliometrics were used for visual analysis. A total of 500 publications with 334 articles and 166 reviews were included, with the timespan of 2012 to 2021. The United States was the most productive country. Majority of the top 10 institutions were from the United States, and Harvard University was the top-contributing institution. The most prolific author and the co-cited author was Thomas N Seyfried from Boston College. The highest cited reference was published in *PLoS ONE*, authored by Abdelwahab Mohammed G, with 161 citations. Glioma and breast cancer were the most common types of cancer in this field, while hepatocellular carcinoma and pancreatic cancer were the new hotspots. The anti-tumor mechanism of KD mainly focused on regulating metabolism, decanoic acid, oxidative stress, fatty acid oxidation, and cell apoptosis. Additionally, the presence of “chemotherapy” and “radiotherapy” in the keywords indicated that KD combined with anti-tumor research was a topic, while “immunotherapy” has became a recent frontiers. Notably, as a metabolic therapy, KD was deserved more attention in the treatment of hepatocellular carcinoma and pancreatic cancer, and KD combined with immunotherapy was the new hotspot and frontier. Additionally, more molecular studies and high-quality uniformly, randomized, controlled clinical trials are urgently warranted to evaluate the effect of KD in multiple cancers.

## Introduction

Cancer is currently the second leading cause of death worldwide, and also placed a heavy burden on health care systems as one of the major public health problems that were difficult to administrate (1). The GLOBOCAN 2020 estimated to account for 19.3 million new cancer cases and almost 10.0 million cancer deaths occurred in 2020 (2). Nowadays, numerous studies have found that diet was closely related to the occurrence and development of cancer (3, 4). Ketogenic diet (KD) consists of a low-carbohydrate combined with high-fat dietary pattern that was first used for the treatment of intractable epilepsy in children (5). Recently, studies has confirmed that KD also maintained an obvious therapeutic effects in a variety of neurological disorders, including, but not limited Alzheimer's and Parkinson's disease, autism, and multiple sclerosis (6–9). Notably, an increasing number of studies were dedicated to the effects of KD on cancer (5, 10), and it has been reported that KD exerted an anti-tumor role by reducing glucose (11), inflammatory response, and oxidative stress (12), inhibiting the mTOR signaling pathway (13), and regulating immune (14).

Bibliometric analysis has been widely used to explore developmental trends and hotspots through quantitative analysis of publications (15). To our knowledge, although the number of publications on KD in the field of cancer were increased steadily, there was no systematic review and bibliometric analysis was performed. Therefore, we visualized the publications with vivid information to (1) identify the most productive, contributors, the key topics, and highly frequent keywords; and (2) to reveal the current research trends of KD on cancer, as well as explore the potential hotspots to shed the light for future research.

## Methods

### Source of data and search strategy

All included data in this study were retrieved from the Web of Science Core Collection (WOSCC) with the following search strings: “Topic = (ketogenic diet) OR (ketogenic diets) OR (ketone diet) OR {[(low carb diet) OR (low carbohydrate diet) OR (carbohydrate-restricted diet)] AND (high fat diet)} and [(neoplasia) OR (neoplasm) OR (neoplasms) OR (carcinoma) OR (tumors) OR (cancer) OR (cancers) OR (malignancy)],” and the retrieved publications were published from January 1, 2012 to December 31, 2021. The detailed search strategies and results were also demonstrated in Table 1. Only original articles and reviews were included; other types, such as editorial materials, letters, and meeting abstracts were excluded. However, there were no geographical restrictions.

**Table 1:** The topic search query.

**Set**	**Results**	**Search query**
#1	7,276	TS = (ketogenic diet) OR (ketogenic diets) OR (ketone diet) OR {[(low carb diet) OR (low carbohydrate diet) OR (carbohydrate-restricted diet)] AND (high fat diet)} Indexes = Web of Science core collection, Time span = 2012–2021
#2	2,510,510	TS = [(neoplasia) OR (neoplasm) OR (neoplasms) OR (carcinoma) OR (tumors) OR (cancer) OR (cancers) OR (malignancy)] Web of Science core collection, Time span = 2012–2021
#3	912	#1 AND #2

### Data collection and analysis

The publication retrieval and data collection were completed on September 2, 2022 to reduce the deviation caused by daily updating of the WOSCC. All the retrieved publications were screened, respectively, by two researchers (RRL and HQC) to ensure their relevance to the topic of this study after the initial data search. Furthermore, the annual number of publications, country/region outputs, journals, citations per paper (CPP), and H-index were also retrieved from the WOSCC. Additionally, impact factors (IF) and quartiles for journal categories were obtained from the Journal Citation Reports 2021 (JCR). Any disagreements were resolved at the discretion of a third senior reviewer (YW).

The website of bibliometrics (http://bibliometric.com/) was applied to generate the visualization cooperation map of the countries/regions, thereby the countries that cooperate most closely with each other were identified. CiteSpace is widely used for visual analysis of publications, which is an excellent choice to identify the current research hotspots and frontiers (16, 17). All data retrieved from WOSCC were exported to CiteSpace software in the mode of “full record and cited references,” which is used to analyze various important bibliometric parameters, including analysis of countries, journals, institutions, authors, references, and a timeline view of cited-references. In addition, keywords co-occurrence clusters and bursts were also captured.

## Results

**Figure 1:**
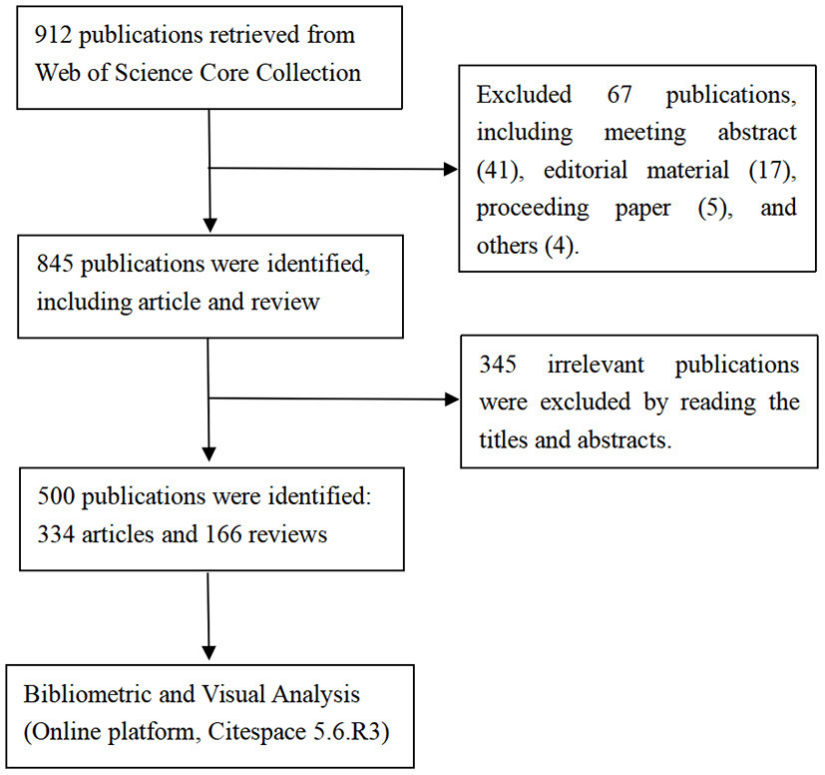
The flowchart of publication screening.

A total of 912 publications in the field of KD on cancer research were retrieved from WOSCC, with the time span of January 1, 2012 to December 31, 2021. Publications that were not articles or reviews were excluded (*n* = 67). Additionally, publications that were unrelated to the research topic (*n* = 345) were also excluded after a careful review of the titles and abstracts by two authors (RRL and CXY). Ultimately, a total of 500 publications were included for final visual analysis, and the specific flowchart of publications screening was shown in Figure 1.

### Annual publication outputs and citations

**Figure 2:**
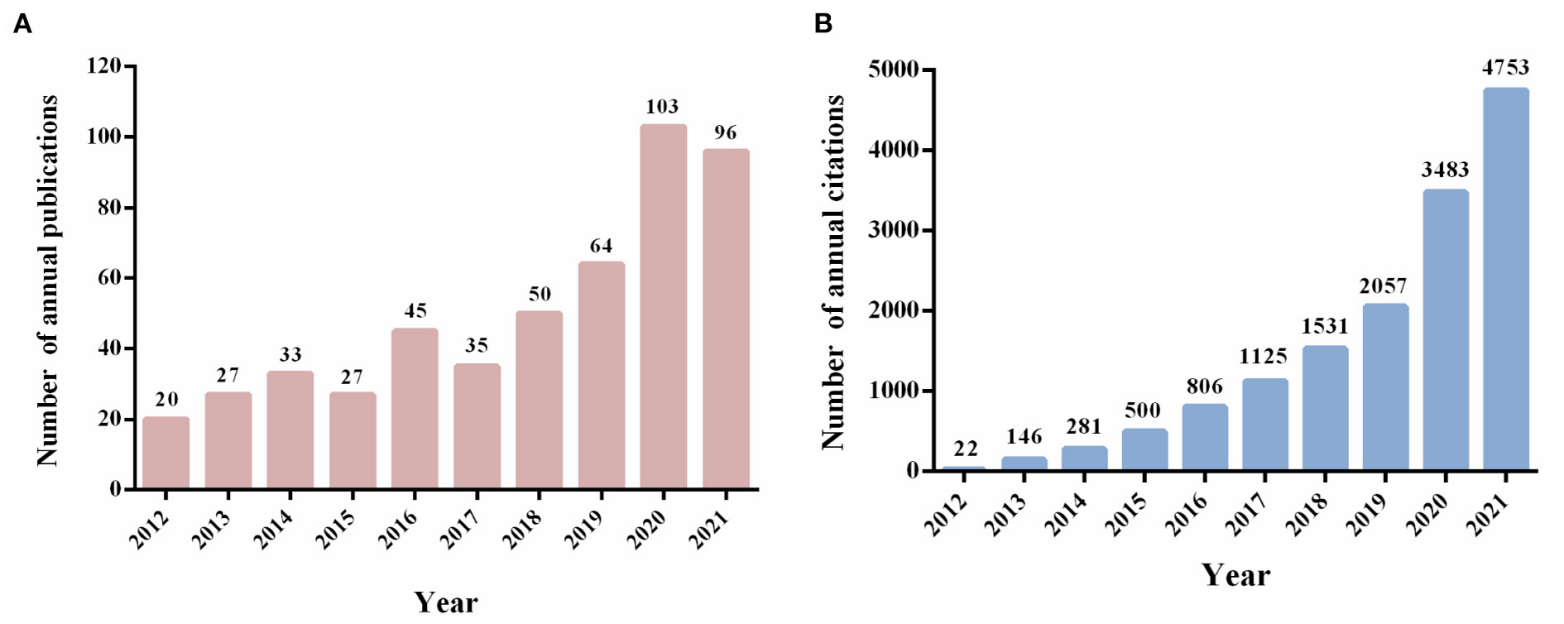
Trends in annual publication outputs and the number of citations in the field of KD on cancer from 2012 to 2021. **(A)** Trends of annual publication outputs. **(B)** Trends of annual citations.

A total of 500 non-duplicate publications, including 334 articles and 166 reviews, were included for bibliometric analysis from 2012 to 2021. The growth of annual publication outputs and annual citations was shown in Figure 2. The number of publications related to KD and cancer had increased steadily in the last decade, whereas there were still some fluctuations between this year. Notably, the number of publications in this field peaked in 2020, with 103 publications, more than five times that of 2012 (*n* = 20) (Figure 2A). As shown in Figure 2B, the number of annual citations had increased significantly over the last decade, reaching the highest point in 2021 (citations = 4,753).

### Distribution of countries/regions and institutions

**Figure 3:**
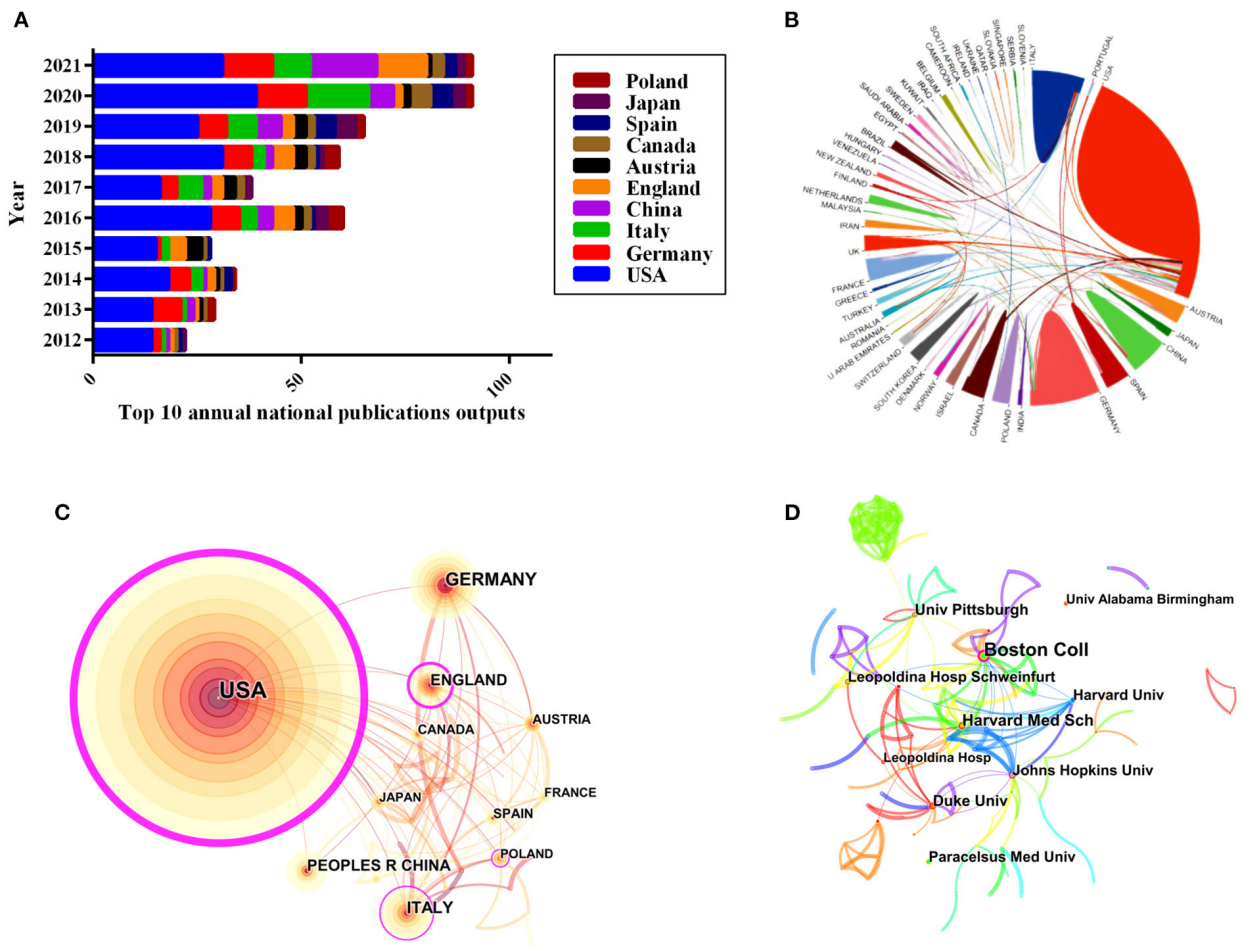
Visualization map of countries/regions and institutions in the field of KD on cancer from 2012 to 2021. **(A)** Number of annual publications and growth trends of the top 10 countries/regions. **(B)** The international network cooperation between the countries, exported the results from the website of bibliometrics (http://bibliometric.com). **(C)** Citespace network map of KD in cancer research involving 57 countries. **(D)** Collaboration network analysis of institutions.

The distribution of all 500 publications covered 57 countries/regions. The top 10 most productive countries/regions were listed in Table 2. The United States was the most prolific country (46.20% of 500, with 231 papers), with the highest number of citations (11,969) and the highest centrality (0.89); followed by Germany (12.80%, with 64 papers), Italy (10.20%, with 51 papers), China (8.00%, with 40 papers), and England (7.60%, with 38 papers). Additionally, the top 10 annual national publication outputs were also identified. The most prolific country of the annual publications was also the United States. Notably, the number of publications in China increased significantly in 2021, surpassing Germany and Italy (Figure 3A). As shown in Figure 3B, the United States and Canada possessed the closest international collaborative in this field, followed by Germany, China, Brazil, and France. The visual analysis of the international cooperation network suggested that 57 countries had established cooperation, with 136 links among one another. Nonetheless, the cooperation was largely centered on the United States, with less cooperation among the remaining countries/regions (Figure 3C).

**Table 2:** The top 10 countries/regions in the field of ketogenic diet on cancer research.

**Rank**	**Countries**	**Counts**	**% of 500**	**Citations**	**H-index**	**Centrality**
1	USA	231	46.20	11,969	58	0.89
2	Germany	64	12.80	1,958	24	0.03
3	Italy	51	10.20	2,870	24	0.14
4	China	40	8.00	813	16	0.01
5	England	38	7.60	2,070	20	0.27
6	Austria	20	4.00	854	14	0.01
7	Canada	20	4.00	638	73	0.07
8	Spain	19	3.80	378	9	0.01
9	Japan	17	3.40	477	10	0.01
10	Poland	17	3.40	382	10	0.18

The top 10 most prolific institutions were shown in Table 3. The leading top 5 institutions of publications were Harvard University with 29 papers, followed by Boston College (18), Pennsylvania Commonwealth System of Higher Education (17), University of California System (17), and Johns Hopkins University (16), respectively. Majority of the top 10 institutions were from the United States. with Harvard University having the highest H-index (20) and the National Institutes of Health ranking the highest number of citations per publication (CPP, 113.15). To further explore the potential relationship between institutions, a visual mapping of institutions was generated by using Citespace. Based on Figure 3D, there were 284 institutions and 335 links, indicating that the distribution of research teams among the institutions was relatively scattered, which shed light on more disciplinary collaboration was urgently needed.

**Table 3:** The top 10 institutions in the field of ketogenic diet on cancer research.

**Rank**	**Institutions**	**Counts**	**% of 500**	**CPP**	**H-index**	**Location**
1	Harvard University	29	5.80	62.76	20	USA
2	Boston College	18	3.60	71.56	15	USA
3	Pennsylvania Commonwealth System of Higher Education	17	3.40	35.71	12	USA
4	University of California System	17	3.40	41.76	11	USA
5	Johns Hopkins University	16	3.20	107.56	12	USA
6	University of Pittsburgh	15	3.00	30.80	10	USA
7	Dana-Farber Cancer Institute	14	2.80	40.21	11	USA
8	Leopoldina Hosp Schweinfurt	14	2.80	33.00	11	France
9	Harvard Medical School	13	2.60	44.08	10	USA
10	National Institutes of Health	13	2.60	113.15	11	USA

### Distribution of journals

The retrieved publications in this study were published in 285 journals, and the top 10 productive journals were shown in Table 4. *Nutrients* was ranked the first in the number of publications (4.80%, with 24 records), followed by *PLoS ONE* (3.60%, with 18 records), *Frontiers in Nutritio* (2.40%, with 12 records), *International Journal of Molecular Sciences* and *Nutrition and Cancer-An International Journal* (1.8%, with 9 records). Notably, the journal of *Cell Metabolism* had both the highest IF (31.372) and CPP (127.50) among the top 10 prolific journals in this field. Further, half of the productive journals had their category quartile in Q1 (Table 4).

**Table 4:** The top 10 scholarly journals in the field of ketogenic diet on cancer research.

**Rank**	**Journal**	**Count**	**% of 500**	**CPP**	**H-index**	**IF (2021)**	**Category Quartile**
1	Nutrients	24	4.80	15.79	11	6.706	Q1
2	PLoS ONE	18	3.60	58.28	15	3.752	Q2
3	Frontiers in Nutrition	12	2.40	11.08	6	6.590	Q1
4	International Journal of Molecular Sciences	9	1.80	24.67	5	6.208	Q2
5	Nutrition and Cancer-An International Journal	9	1.80	15.00	5	2.816	Q4
6	Journal of Neuro-Oncology	8	1.60	36.00	7	4.506	Q2
7	BMC Cancer	7	1.40	34.29	6	4.638	Q2
8	Journal of Translational Medicine	7	1.40	90.43	6	8.440	Q1
9	Clinical Nutrition	6	1.20	14.33	6	7.643	Q1
10	Cell Metabolism	6	1.20	127.50	6	31.372	Q1

### Distribution of authors and cited-authors

**Figure 4:**
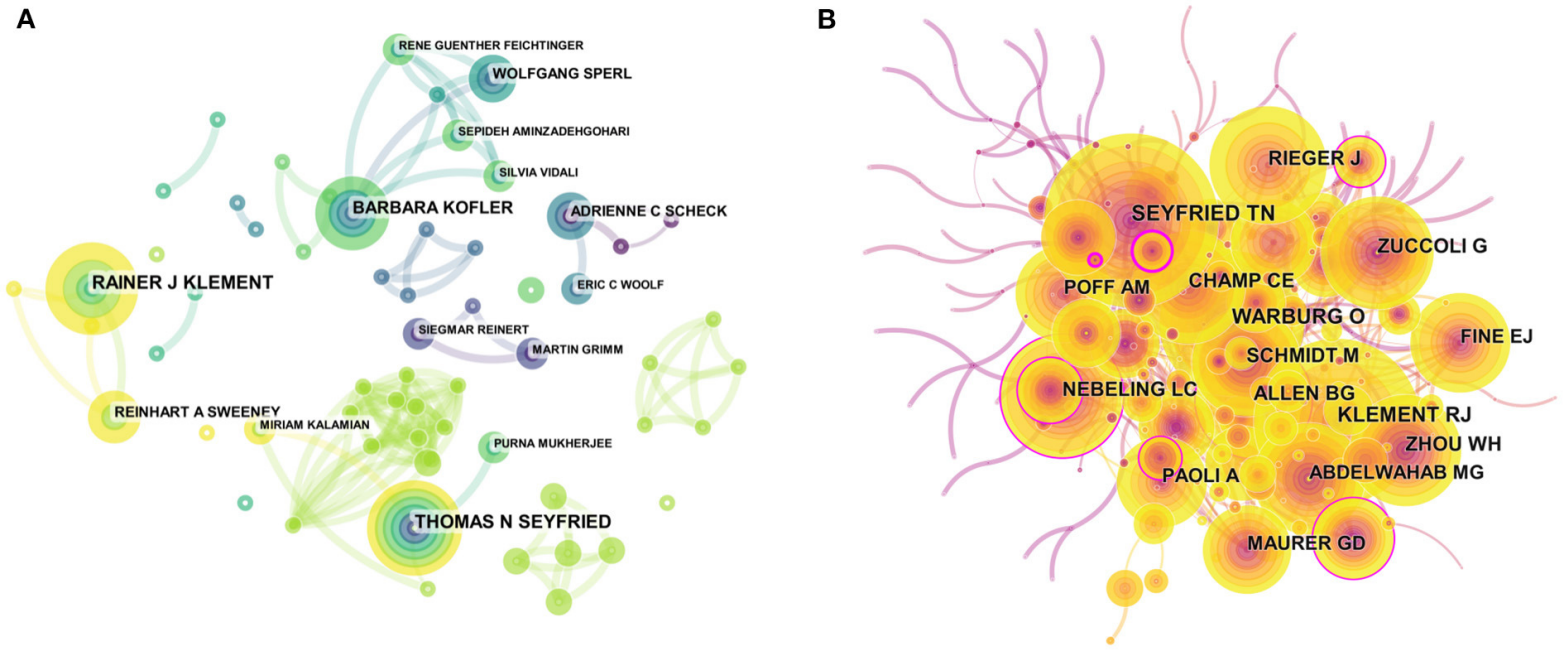
Visualization map of authors/cited-authors. **(A)** Visualization map of authors. **(B)** Visualization map of cited-authors. Nodes indicate authors, and the lines between nodes indicate collaborative relationships. The larger the node area, the greater the number of publications by that author or cited author.

The visual map of authors consisted of 314 nodes by using Citespace. The top 10 most prolific authors were shown in Table 5. Thomas N Seyfried was the most prolific author, with 12 publications, followed by Rainer J Klement (11), Barbara Kofler (9), Adrienne C Scheck (6), and Reinhart A Sweeney (6), respectively. The collaboration of 105 authors was demonstrated in Figure 4A. Furthermore, the network visualization of co-cited authors was shown in Figure 4B. The largest nodes were related to the authors with the highest number of collaborations, including Thomas N Seyfried (140 citations), Rainer J Klement (133 citations), Warburg O (118 citations), Nebeling LC (96 citations), and Rieger J (94 citations) (Table 5). Interestingly, Thomas N Seyfried and Rainer J Klement were the authors with the highest number of publications and co-citations, which indicated that they may be core researchers in the field of KD and cancer research.

**Table 5:** The top 10 authors and co-cited authors in the field of ketogenic diet on cancer research.

**Rank**	**Author**	**Count**	**Rank**	**Co-cited author**	**Frequency**
1	Thomas N Seyfried	12	1	Thomas N Seyfried	140
2	Rainer J Klement	11	2	Rainer J Klement	133
3	Barbara Kofler	9	3	Warburg O	118
4	Adrienne C Scheck	6	4	Nebeling LC	101
5	Reinhart A Sweeney	6	5	Rieger J	96
6	Wolfgang Sperl	4	6	Allen BG	94
7	Miriam Kalamian	4	7	Champ CE	93
8	Siegmar Reinert	4	8	Zhou WH	92
9	Martin Grimm	4	9	Zuccoli G	92
10	Eric C Woolf	4	10	Schmidt M	91

### Analysis of cited-references

**Figure 5:**
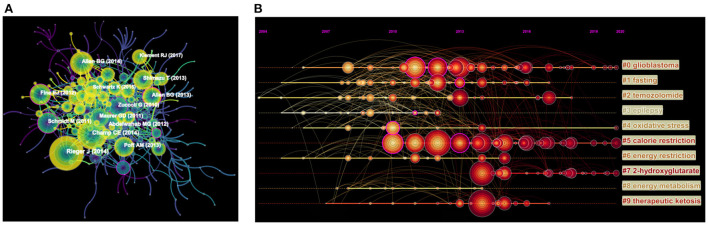
**(A)** Network visualization map of cited-references in the field of KD on cancer research. The nodes represent cited-references, and the lines between the nodes represent cited-references relationships. **(B)** Timeline view of co-cited references in the field of KD on cancer research. The cluster with warmer color and larger nodes contained more publications, indicating that this clustering issue was the hotspot in this field.

The visual map of cited-references consisted of 507 nodes and 2,674 links (Figure 5A). The characteristics of the top 10 highest cited-references were shown in Table 6. Based on Table 6, 5 of the top 10 highest cited-references were related to basic and clinical research on glioma, which indicated that research on glioma has been a hotspot in this field. Among them, entitled “*The Ketogenic Diet Is an Effective Adjuvant to Radiation Therapy for the Treatment of Malignant Glioma*” was published by Abdelwahab, MG, with the highest frequency of citations, suggesting that KD could significantly enhance the anti-tumor effect of radiotherapy, and pointed out that KD-induced cellular metabolism may be the current adjuvant treatment standard for malignant gliomas (18). In addition, the article entitled “*Ketogenic Diets Enhance Oxidative Stress and Radio-Chemo-Therapy Responses in Lung Cancer Xenografts*” published in *Clinical Cancer Research* (with IF 13.801), revealed that KD can improve the chemoradiotherapy response of lung cancer by modulating oxidative stress and inhibiting the proliferation of cancer cells (19).

**Table 6:** The top 10 high-cited references in the field of the ketogenic diet on cancer research.

**Rank**	**References**	**Journal**	**IF2021**	**First author**	**Publication time**	**Total citations**
1	The Ketogenic Diet Is an Effective Adjuvant to Radiation Therapy for the Treatment of Malignant Glioma	PLoS ONE	3.752	Abdelwahab, Mohammed G	2012	161
2	Metabolic management of glioblastoma multiforme using standard therapy together with a restricted ketogenic diet: Case Report	Nutrition & Metabolism	4.654	Zuccoli, Giulio	2010	161
3	Ketogenic diets as an adjuvant cancer therapy: History and potential mechanism	Redox Biology	10.787	Allen, Bryan G	2014	155
4	A pilot study of ketogenic diet in recurrent glioblastoma	International Journal of Oncology	5.884	Rieger, Johannes	2014	153
5	Effects of a ketogenic diet on the quality of life in 16 patients with advanced cancer: A pilot trial	Nutrition & Metabolism	4.654	Schmidt, Melanie	2011	151
6	Differential utilization of ketone bodies by neurons and glioma cell lines: a rationale for ketogenic diet as experimental glioma therapy	BMC Cancer	4.638	Maurer, Gabriele D	2011	131
7	Targeting metabolism with a ketogenic diet during the treatment of glioblastoma multiforme	Journal of Neuro-Oncology	4.506	Champ, Colin E	2014	123
8	Ketogenic Diets Enhance Oxidative Stress and Radio-Chemo-Therapy Responses in Lung Cancer Xenografts	Clinical Cancer Research	13.801	Allen, Bryan G	2013	120
9	The Ketogenic Diet and Hyperbaric Oxygen Therapy Prolong Survival in Mice with Systemic Metastatic Cancer	Plos One	3.752	Poff, Angela M	2013	104
10	Beneficial effects of ketogenic diets for cancer patients: a realist review with focus on evidence and confirmation	Medical Oncology	3.738	Klement, Rainer J	2017	79

The timeline view of the cited-references in the field of KD on cancer research was generated by using Citespace (Figure 5B), with the bold timeline indicating that the clustering topic was a hotspot during this period. As shown in Figure 5B, 9 cluster labels were extracted from the cited-references using the log-likelihood rate method, among which glioblastoma (cluster #0) and temozolomide (cluster #2) were closely related; while fasting (cluster #1), oxidative stress (cluster #4), and 2-hydroxyglutarate (cluster #7) may reveal the potential mechanism of KD in cancer treatment.

### Analysis of keywords, co-occurrence clusters and burst

**Figure 6:**
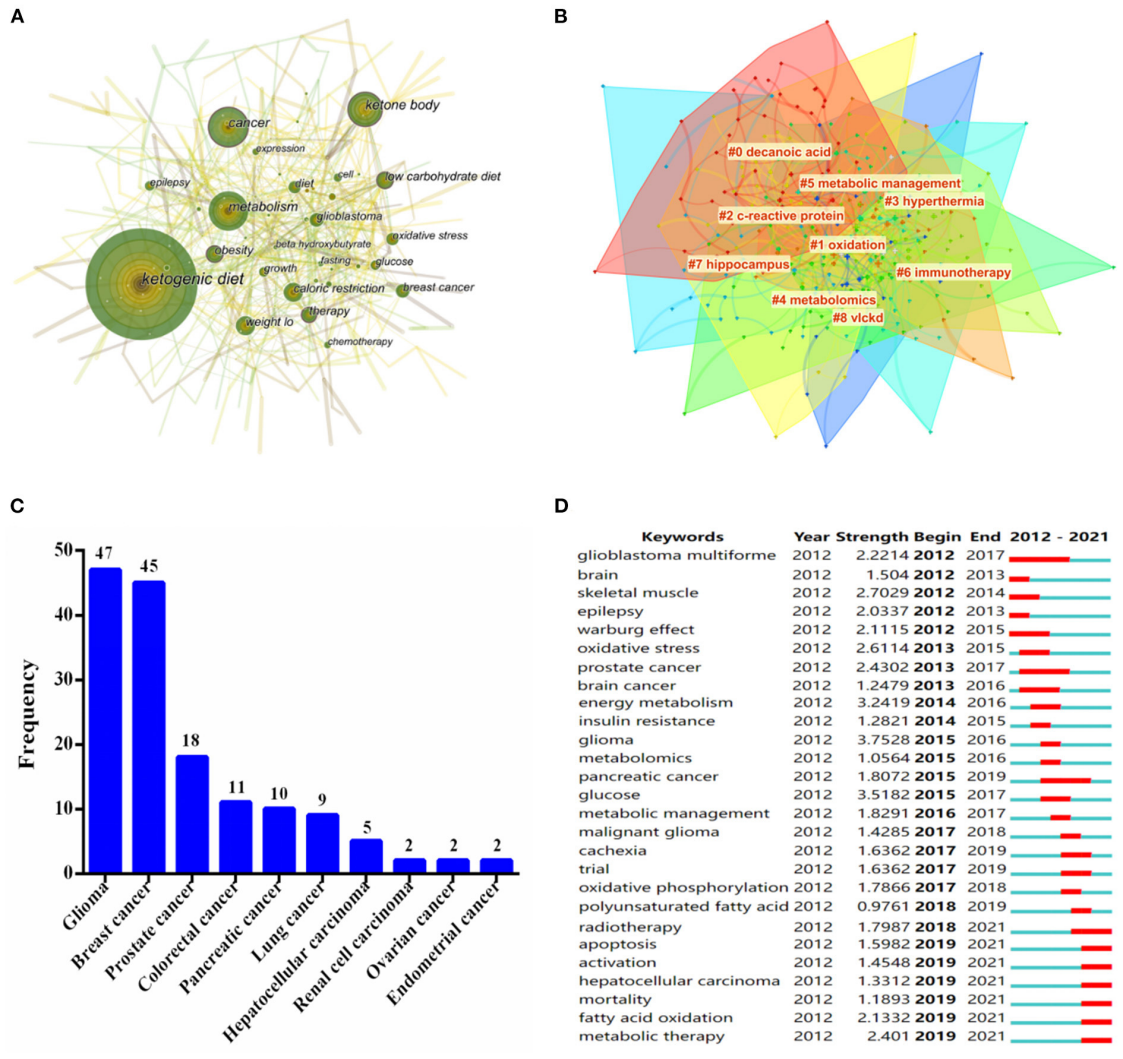
**(A)** Map of keywords occurrence in the field of KD on cancer research from 2012 to 2021. **(B)** The cluster of keywords in the field of KD on cancer research. The different colors mean different clusters. **(C)** Top 10 cancer types in the field of KD on cancer research. **(D)** Keywords with periods of the strongest burst from 2012 to 2021 among the top 25 burst keywords in publications in the field of KD on cancer research.

A total of 21 keywords were identified as occurring more than 25 times (Figure 6A). Notably, “metabolism,” “ketone body,” and “caloric restriction” were the keywords that appeared most frequently in this field over the past 10 years, except for the topic terms of “ketogenic diet” and “cancer.” In addition, a visual map of keywords co-occurrence clusters mainly included but not limited to the following aspects: cluster #0 named “decanoic acid,” was the largest cluster, followed by “oxidation,” “c-reactive protein,” “hyperthermia,” “metabolomics” and “immunotherapy” (Figure 6B).

To our interest, the application of KD in various types of cancers over the past decade was summarized by further analyzing of the keywords. As shown in Figure 6C, glioma had become a research hotspot in this field, with a frequency of 47, followed by breast cancer (45), prostate cancer (18), colorectal cancer (11), and pancreatic cancer (10), respectively. This finding indicated that the earliest application of KD in the treatment of glioma, while it was also crucial in the treatment of breast cancer or other types of cancers.

Specifically, keywords burst detection was executed to acquire a brisk sight of future research trends in the field of KD on cancer (Figure 6D). The red line indicated a sudden burst of keywords during the relevant period, whereas the blue line means unpopularity. Figure 6D demonstrate that glioblastoma multiforme was the earliest research hotspot in the field of KD on cancer, spanning the years 2012 to 2017, with a burst strength of 2.2214. When focusing on the keywords after 2019, the strongest burst keywords were “apoptosis,” “activation,” “hepatocellular carcinoma,” “mortality,” “fatty acid oxidation,” and “metabolic therapy,” respectively.

## Discussion

### General information

This study is the first bibliometric and visual analysis for the research of KD on cancer. In this study, a total of 500 publications were included, with 334 articles and 166 reviews, spanning the period from 2012 to 2021. Publications on KD and cancer research showed an upward trend over the last decade, especially in the last 2 years, with the numbers of publications remaining around 100, indicating that this field is receiving increasing attention from the researchers. A total of 57 countries/regions and 314 authors contributed to this field.

The most productive country was the United States, with the highest number of citations, centrality, and annual publications. Meanwhile, most of the top 10 institutions were from the United States, with the leading institutions were Harvard University, Boston College, Pennsylvania Commonwealth System of Higher Education, and the University of California System, respectively, indicating that the United States was the main driver of the highest academic reputation in this field. Additionally, Germany, Italy, the People's Republic of China, and England excited a certain influence in this field, which was confirmed by the following characteristics: number of publications, number of citations, and the H-index values. Notably, the number of publications from China increased significantly in 2021, surpassing Germany and Italy, which indicated that Chinese scholars have paid more attention to this field in recent years.

All publications were published in 285 journals, with the leading journals including *Nutrients, PLoS ONE*, and *Frontiers in Nutrition*. Interestingly, Thomas N Seyfried from Boston College was the most prolific author and the most co-cited author, suggesting that the author had a high level of achievement in this field. Additionally, among the top 10 highly cited references in the field of KD on cancer research, we found that more than half of them reported on KD for malignant glioma. The highest cited reference was published in *PLoS ONE*, and authored by Abdelwahab Mohammed G with 161 citations, which reported that KD enhanced the anti-tumor effect of radiotherapy in malignant gliomas by elevating the level of β-hydroxybutyrate (18). Furthermore, “malignant glioma” was the most common type of cancer in this field, which may be closely related to the first use of KD in the treatment of brain glioma.

### Hotspots and development trends

The hotspots and frontiers in this field could be revealed by the highest cited references, analysis of keyword burst and co-occurrence clusters. By generalizing the analysis, we can draw the following points in this field. First, the hotspots and trends of KD in cancer types. The keywords “hippocampus,” “glioblastoma” and “temozolomide” from the clusters of keywords and co-cited references indicated that the research of KD on cancer was mainly focused on glioma. As well-known, KD was first used in epilepsy treatment for effective symptoms control (20). Meanwhile, one in four patients undergoing epilepsy surgery has brain tumors, indicating a strong connection was existed between the brain tumors and epilepsy (21). In addition, keywords analysis demonstrated that KD has high attention on the research of breast cancer and prostate cancer. A recent clinical study demonstrated that KD therapy played a beneficial role by reducing TNF- α, and insulin, and increasing IL-10 in breast cancer patients (22). Besides, Kaiser et al. concluded that KD could inhibit the growth of prostate cancer through regulation of the insulin resistance signaling pathways (23). Notably, the burst keywords of “hepatocellular carcinoma” and “pancreatic cancer” indicated that the treatment of KD in the two types of cancer were the new hotspots. Wang et al. reported that KD could inhibit the progression of HCC by increasing the expression of hydroxymethylglutaryl-CoA synthase 2 in hepatocytes and regulating lipid metabolism (24). Meanwhile, KD exerted anti-HCC effects and increased serum β-hydroxybutyrate levels by inducing histone hyperacetylation (25). Summarily, liver is the glucose and lipid metabolic center that produces ketone bodies, KD has a broad application prospect in the treatment of HCC (26). In addition, several recent studies have demonstrated that KD effectively inhibited the proliferation of pancreatic by reprogramming cell metabolism, epigenome, and the gut microbiome (27, 28). These results suggested that glioma and breast cancer were the most studied cancer types in this field, while hepatocellular carcinoma and pancreatic cancer were the new hotspots and trends.

Second, the hotspots and trends of the anti-tumor mechanism of KD. Through the analysis of keywords and cited-references, we found that the anti-tumor mechanism of KD mainly focused on regulation of metabolism, decanoic acid, fatty acid oxidation, oxidative stress, and apoptosis. Robust evidence showed that KD executed anti-tumor effect by regulating metabolism, which is manifested by a decrease in glucose concentration and an increase in the concentration of ketone body and 3-hydroxybutyric acid (29, 30). Ketone body and 3-hydroxybutyric acid were confirmed to play an important role in promoting tumor immunity by inhibiting the expression of NLRP3 inflammasome (31). Cancer cells were mainly metabolized using aerobic glycolysis (Warburg effect), nevertheless KD as a carbohydrate-restricted dietary pattern, can effectively inhibit glucose/insulin signaling, mitochondrial metabolism and inflammation reaction, thereby hindering the development and deterioration of cancers (32, 33). Notably, the latest study found that KD could promote the production of decanoic acid, and inhibit the AMPA receptor, given that to inhibit the growth of cancer (34). Unfortunately, oxidative stress may aggravate both the damage of tissue and the progression of cancer *via* promoting the formation of reactive lipid fragments (35). Nevertheless, oxidative stress was significantly inhibited by KD therapy to contribute the effects of anti-inflammation and anti-tumor properties (36). Meanwhile, the metabolism regulation of KD to exert anti-tumor effects has been widely researched, while elucidating the anti-tumor effects of KD from the perspective of decanoic acid and fatty acid oxidation is a current hotspot.

Third, the hotspots and trends of KD synergy with anti-tumor. Based on the analysis of keywords, several keywords related to anti-tumor treatment, such as chemotherapy, radiotherapy, and temozolomide were identified, indicating that KD combined with anti-tumor therapy is a hotspot in this field. Further study confirmed that breast cancer patients in the KD combined with chemotherapy group had a higher quality of life and physical activity scores at 6 weeks compared to the chemotherapy group, while lower levels of lactate and ALP in serum, suggesting that KD may significant beneficial for the patients with breast cancer (37). Simultaneously, accumulating evidence suggested that KD therapy can improve chemotherapy sensitivity and reduce target lesions in different cancers, such as lung cancer, pancreatic cancer, and gastric cancer (38–41). In addition, radiotherapy is the mainstay of anti-tumor treatment, which can significantly improve the survival rate of cancer patients. Study had reported that KD preserved fat free and skeletal muscle mass and effectively improved global quality of life in breast cancer patients undergoing curative radiotherapy (13); it also partially counteracted the adverse effects of radiotherapy on body composition in head and neck cancer patients (42). Importantly, the cluster of keywords showed that immunotherapy is the new hotspot in this field recently. Ferrere G confirmed that KD enhanced the anti-neoplastic effects of PD-1 blockade (43). Further, the latest experiment reinforced theory that KD partly stimulated anti-tumor immunity and strengthened checkpoint blockade immune-therapy *via* AMPK mediated decreasing PD-L1 protein abundance (14). To the best of our knowledge, most of studies had focused on animal experiments in this field, whereas the robust clinical evidence was still inadequacy. Hence, more high-quality randomized clinical trials to evaluate the combined effects of KD and anti-neoplastic therapy, especially the combination of KD and immunotherapy, are urgently warranted, which is the current research trend in this field. Notably, although KD was regarded as a tolerable method during the clinical management of cancers, some adverse effects have also been reported, such as causing constipation, fatigue, and micronutrient deficiencies in the patients with cancers (44, 45). Therefore, more attention should be paid to the potential adverse reactions of KD in the treatment of anti-tumor in the future clinical researches.

### Strengths and limitations

This is the first bibliometric and visual analysis in the field of KD on Cancer, and this study identified the latest research trends and hotspots in this field. Hence, a perspective to the developing trend of KD on cancer was presented, which may help researchers to explore new directions for future research. However, some limitations should be illustrated. First, the data were only extracted from the WOSCC, and no other databases (such as PubMed and Embase) were used, which may lead to under-extraction of publications. However, WOSCC has collected more than 20,000 authoritative and influential academic journals. Therefore, the results and findings of this study were robust. Second, only studies published in English were retrieved from the database, which may lead to source bias. In addition, although we have clearly defined the research topic during the search, it is still not guaranteed that every document is completely relevant to the topic, which leads to the results of incomplete inclusion of core keywords. However, incorrect extraction and omission of publications could be prevented by topic searches. Therefore, this study can still highlight the general situation and research trends in this field.

## Conclusions

This study provided a general overview of the main research hotspots and frontiers of KD on cancer research. Summarially, glioma and breast cancer were the most studied cancer types in this field, while hepatocellular carcinoma and pancreatic cancer gained more attention recently. In addition, KD may exert anti-tumor effects by regulating metabolism, oxidative stress, and fatty acid oxidation. Meanwhile, current clinical studies mainly focused on KD combined with chemoradiotherapy for glioma and breast cancer, and the findings demonstrated that KD could effectively improve the anti-tumor effects, reduce adverse effects, and enhance the quality of life. However, more molecular studies and corresponding large-sample RCTs are urgently needed to assess the clinical application of KD as a metabolic therapy, and the potential adverse effects of KD in cancer treatment should be closely concerned. Meanwhile, the development of diversified ketogenic products to improve patient compliance is also a hotspot for future research due to the difficulty of long-term adherence to KD in cancer patients.

## Data availability statement

The raw data supporting the conclusions of this article will be made available by the authors, without undue reservation.

## Author contributions

RL, YW, JF, and YL designed the study. RL, QH, and CY were responsible for data collection and drafted the manuscript. RL, QH, CY, CW, and NL were responsible for investigation, figures, and tables construction. YW, JF, and YL revised and approved the final version of the manuscript. All authors contributed to the article and approved the submitted version.

## Funding

This study was supported by Traditional Chinese Medicine Research Program of Zhejiang Province (No. 2018ZQ020); Medical and Health Platform Program of Zhejiang Province (No. 2021KY556).

## Conflict of interest

The authors declare that the research was conducted in the absence of any commercial or financial relationships that could be construed as a potential conflict of interest.

## Publisher's note

All claims expressed in this article are solely those of the authors and do not necessarily represent those of their affiliated organizations, or those of the publisher, the editors and the reviewers. Any product that may be evaluated in this article, or claim that may be made by its manufacturer, is not guaranteed or endorsed by the publisher.
